# Case Report: Lessons Learned From Aortic Valve Rupture After Blunt Chest Trauma

**DOI:** 10.3389/fped.2021.660519

**Published:** 2021-05-14

**Authors:** Qu-ming Zhao, Ling-yu Lai, Lan He, Fang Liu

**Affiliations:** ^1^Pediatric Heart Center, Children's Hospital of Fudan University, National Children's Medical Center, Shanghai, China; ^2^General Pediatric Ward, Children's Hospital of Fudan University, National Children's Medical Center, Shanghai, China

**Keywords:** blunt chest trauma, aortic valve rupture, heart failure, echocardiagraphy, aortic regurgitation

## Abstract

Aortic valve rupture (AVR) due to blunt chest trauma is extremely rare in the pediatric population, and little attention has been paid to such damages. Early diagnosis of AVR may not be easy in patients with multiple competing injuries and poor acoustic windows. We report a case of delayed diagnosis of AVR in a 12-year-old boy after falling from a height of 15 meters, who presented with recurrent hemoptysis and ventilator dependence. This rare case highlights the importance of performing transesophageal echocardiography in trauma patients when the images of transthoracic echocardiography are suboptimal, especially for those presenting with signs and symptoms suggestive of heart failure. The overall prognosis of aortic valve replacement is good.

## Introduction

Traumatic injury remains the primary cause of mortality in the pediatric population, and the incidence of blunt chest trauma (BCT) has continuously risen in the past few years (1), mainly owing to increasing numbers of automobile accidents or falls from a height. The cardiac injury occurs in up to 76% of BCT patients (2), and the predominant types are wall rupture and contusion, while valve injury is infrequent, affecting only 0.85% of the pediatric cardiac injury population (3). Although the aortic valve is the most commonly injured valve, only one case of aortic valve rupture (AVR) was seen in an autopsy series of 546 deaths due to BCT (4), and only 95 cases of aortic regurgitation (AR) after BCT have been reported all over the world up to 2015 (5). Traumatic AVR is even more unusual in the pediatric literature, with only 5 cases reported, and the youngest was 15 years of age (5). Thus, timely diagnosis of AVR may not be easy at the initial assessment if the manifestations of AR were masked by the consequences of coexistent injuries, especially when cardiac involvement is not suspected. Here We report a case of delayed diagnosis of AVR after falling from a height of 15 meters, who presented with recurrent hemoptysis and ventilator dependence.

## Case Description

A previously healthy 12-year-old boy was transferred to our institution from a local hospital on the 3rd day after falling from a height of 15 meters. Chest X-ray and whole-body computed tomography (CT) scan on the day of the injury showed multiple traumas, including pulmonary contusion, renal injury, and multiple rib fractures. The following day, two blood cultures were taken because of high fever (39.3°C) and elevated inflammatory markers, including C-reactive protein (CRP) (45 mg/L), erythrocyte sedimentation rate (ESR) (70 mm/h), and leukocytosis (19,300/mm^3^). The patient was treated with cefotaxime for presumed sepsis but had no clinical improvement. Nevertheless, according to the referral notes, the patient's vital signs were normal except for tachycardia (130 bpm).

**Figure 1:**
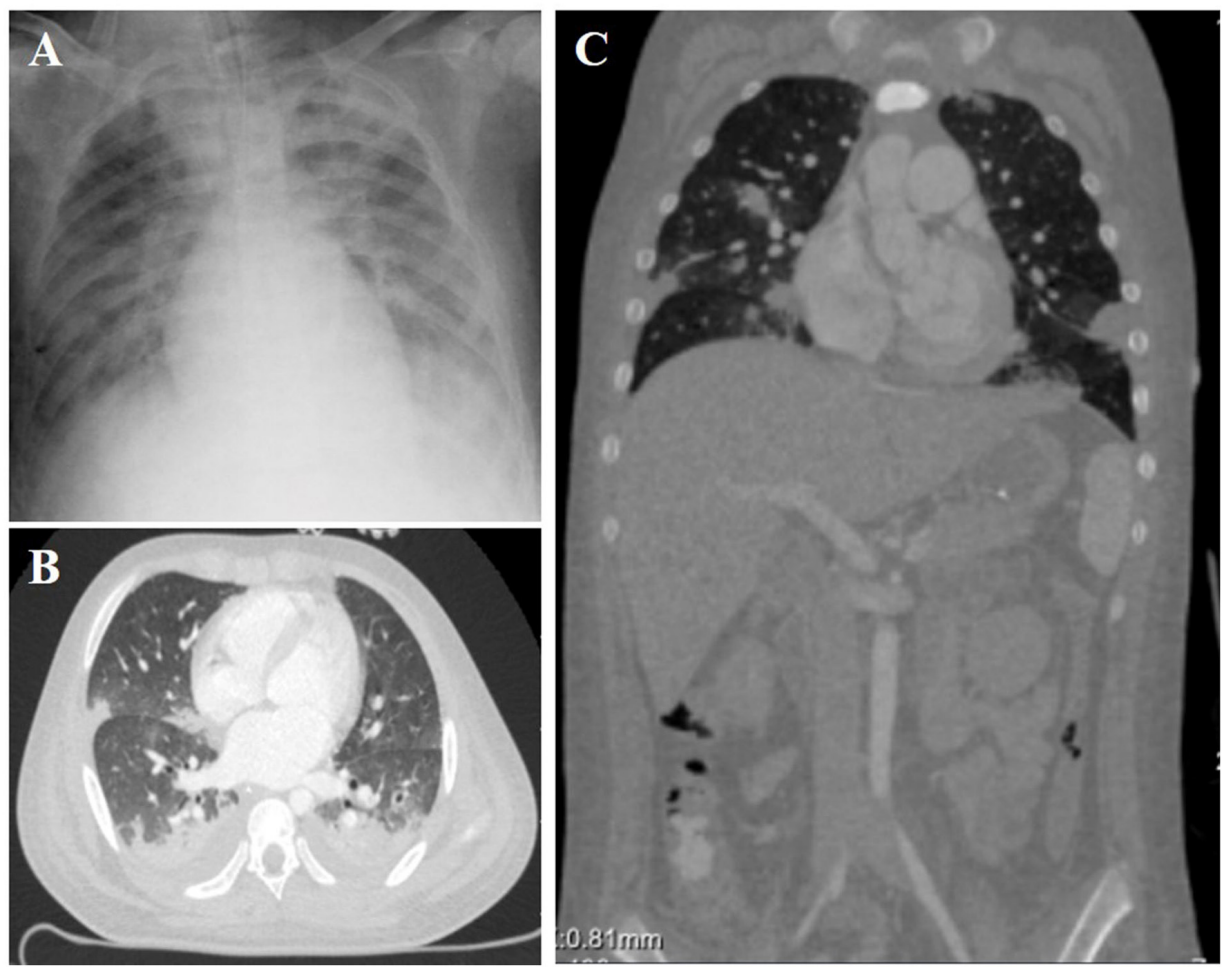
Chest X-ray **(A)** and Contrast-enhanced computed tomography **(B,C)** showed mild cardiomegaly, interstitial pulmonary edema, and bilateral pulmonary contusions.

When he arrived at our institution, he required mechanical ventilation for progressively worsening dyspnea. A widened pulse pressure was noted (120/40 mmHg), which the critical care physician considered to be a manifestation of intracranial or spine injuries. He had a regular heart rate of 120 bpm, while his respiratory rate and oxygen saturations were normal under mechanical ventilation. Physical examination revealed moist rales over both lungs, a heart murmur that was difficult to characterize due to the tachycardia, and no abdominal or pelvic mass was detected on palpation. On the day of admission, bilateral pulmonary infiltrates and cardiomegaly were noted on the chest X-ray (Figure 1A). A contrast-enhanced CT scan showed pulmonary contusions, hemopneumothorax, renal laceration, and multiple bone fractures, including ribs, pelvis and lumbar vertebra. Cardiac severe injuries, such as aortic dissection, aortic aneurysm, and cardiac tamponade, were not found (Figures 1B,C). Initial transthoracic echocardiography by portable ultrasound machine revealed a functionally normal heart. However, the anatomy of cardiac valves and color Doppler flow images not well-visualized because of the patient's body habitus and hemopneumothorax. Based on suggestions from multidisciplinary consultation, conservative treatment, including respiratory support, anti-infection, and external fixation to stabilize the fracture., was preferred for this patient.

**Figure 2:**
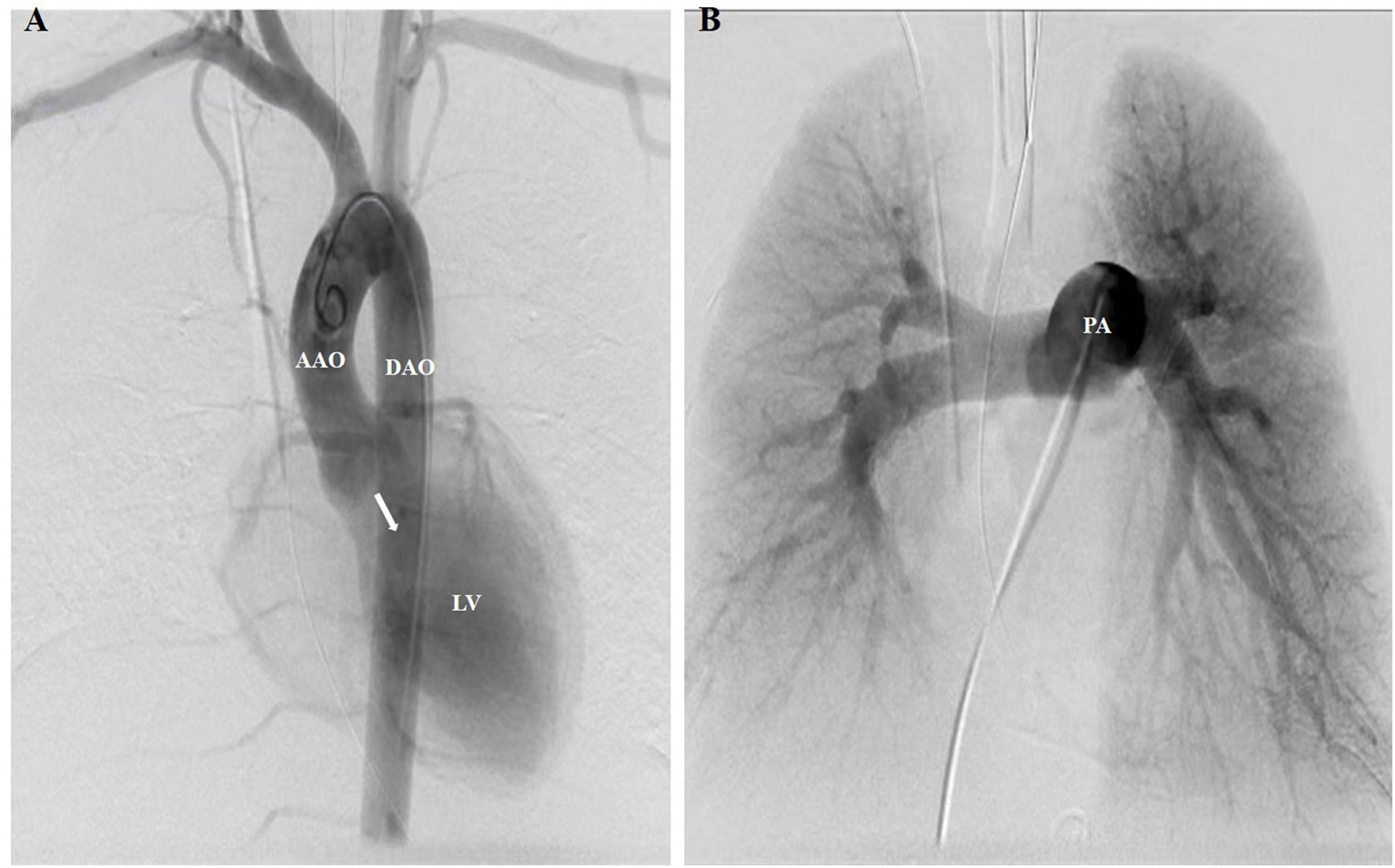
A pigtail catheter was advanced for the aortic and pulmonary angiogram. **(A)** Aortic angiogram showed severe diastolic retrograde flow (white arrow) from the AAO to the LV. No enlarged or ruptured bronchial arteries, which may be responsible for hemoptysis, were found to originate from the supra-aortic branches or DAO. **(B)** Pulmonary angiogram showed no pulmonary vascular abnormalities responsible for hemoptysis, such as post-traumatic PA pseudoaneurysms and pulmonary arteriovenous malformations. AAO, ascending aorta; DAO, descending aorta; LV, left ventricle; PA, pulmonary artery.

**Figure 3:**
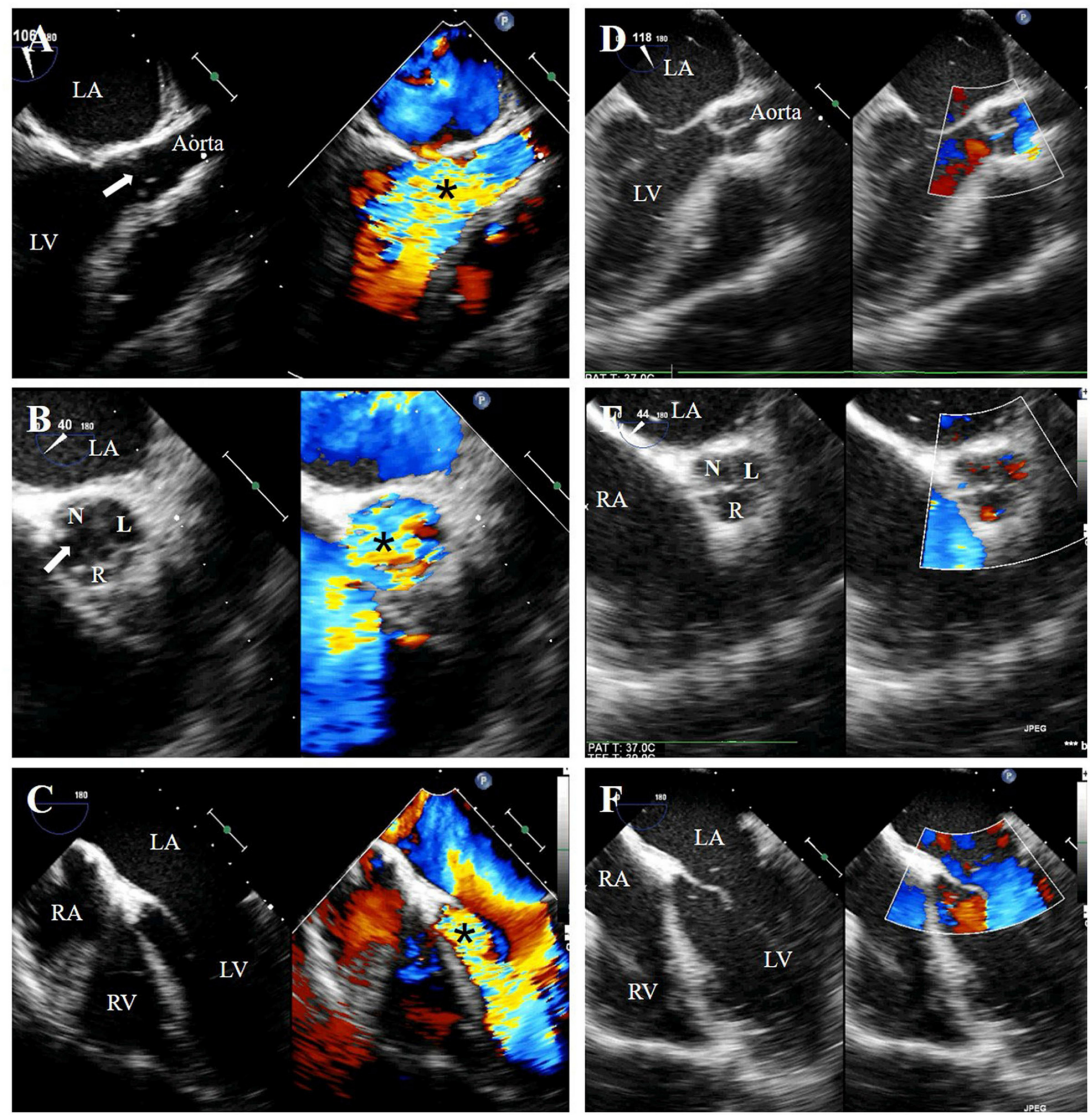
Preoperative transesophageal echocardiography (TEE) showed free-flow aortic regurgitation (black asterisk), which is caused by a fissure (white arrow) formed between the flailed right coronary cusp and non-coronary cusp, on the long-axis view **(A)**, short-axis view **(B)** and five-chamber view **(C)**. Postoperative TEE showed well-functioning new valves without regurgitation **(D–F)**. N, non-coronary cusp; R, right coronary cusp; L, left coronary cusp; LA, left atrium; RA, right atrium; LV, left ventricle; RV, right ventricle.

On the 2nd day after admission, the inflammatory markers remained elevated (CRP 66 mg/L, ESR 90 mm/h, and leukocytosis 21,300/mm^3^), and blood cultures yielded meropenem-susceptible pseudomonas aeruginosa. Mild increases in myocardial injury biomarkers, including cardiac troponin I (CTnI) (2.5 ng/ml), creatine kinase MB (CKMB) (120 U/L) and N-terminal of prohormone brain natriuretic peptide (NT-pro BNP) (750 pg/ml), were considered common in BCT and had not been paid adequate attention. After the administration of sensitive antibiotics, his fever did not abate until 14 days after admission, by which time his blood cultures had turned negative and inflammatory markers had returned to normal. Contradictory to the improvement in sepsis and pulmonary contusions, he developed recurrent hemoptysis on admission day 17 and could not be weaned from the ventilator. His diastolic blood pressure remained low (30–45 mmHg) and myocardial injury biomarkers (CTnI 3.42 ng/ml, CKMB 301 U/L and NT-pro BNP 14,376 pg/ml) were getting higher despite multiple measurements of normal left ventricular ejection fraction(65–75%). Repeated chest radiographs suggested pulmonary edema. Catheter angiography was intentionally performed on day 21 after admission to rule out the post-traumatic pulmonary artery pseudoaneurysms and ruptured bronchial artery aneurysm. However, the aortic angiogram unexpectedly showed wide-open aortic insufficiency (Figure 2). A transesophageal echocardiogram (TEE) was then obtained and revealed mild left heart dilatation and severe eccentric aortic regurgitation due to flail non-coronary cusp (NCC) and right coronary cusp (RCC) (Figures 3A–C). Following confirmed diagnosis, he was noted to have a 2/6 diastolic murmur at the left lower sternal border and a 2/6 systolic murmur at the right upper sternal border by a cardiologist.

**Figure 4:**
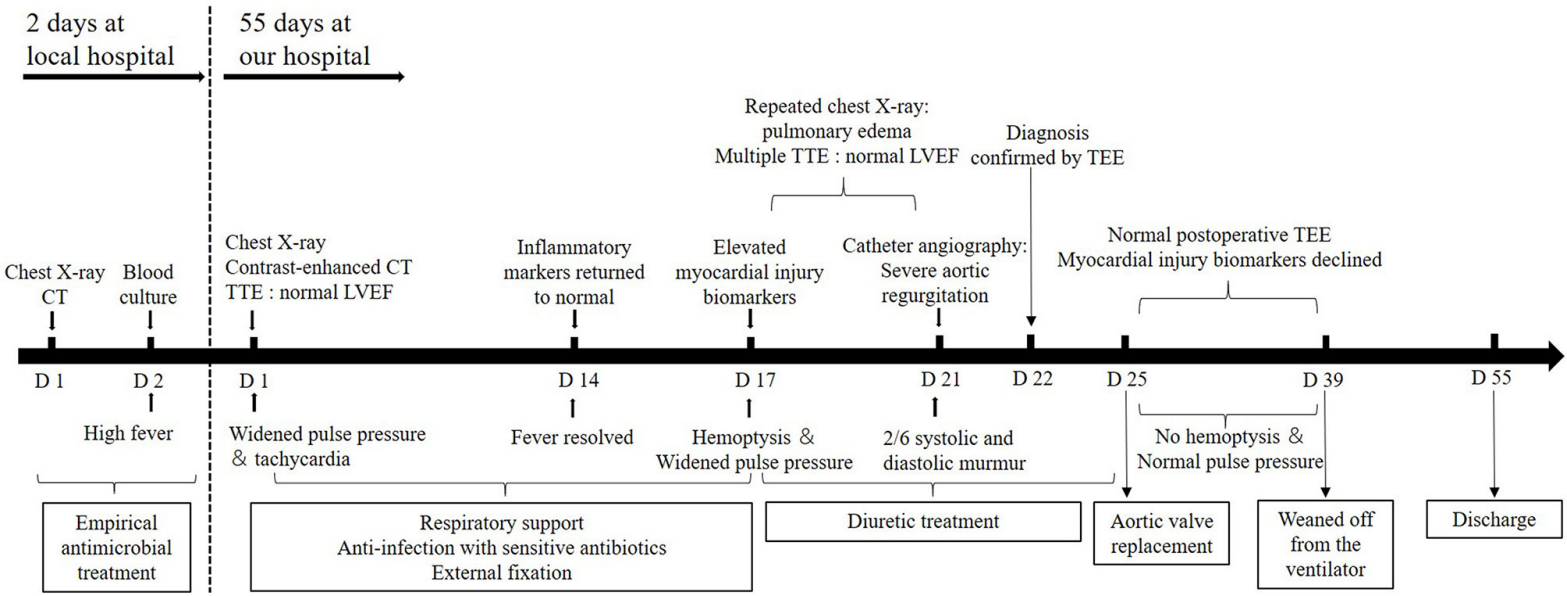
A timeline of the patient's diagnosis and treatment process. CT, computed tomography; TTE, transthoracic echocardiography; TEE, transesophageal echocardiography; LVEF, left ventricular ejection fraction.

The patient underwent cardiac surgery on admission day 25 after diuretic treatment. The intraoperative view revealed the morphologically normal NCC and RCC were torn entirely off from the annulus. The damaged valves were then excised and replaced with bovine pericardium tissue valves. Histopathological examination of the valves demonstrated no evidence of any inflammation. Postoperative TEE showed well-functioning new valves without regurgitation (Figures 3D–F). His blood pressure returned to normal in the immediate post-operative period (110/70 mmHg). The levels of CTnI, CKMB, and NT-pro BNP gradually declined and reached near the baseline level on post-operative day 10. He had no recurrent hemoptysis but had a prolonged intensive care unit stay due to ventilator-associated pneumonia. He was weaned off from the ventilator 14 days after the surgery and was ultimately discharged to a rehabilitation facility in stable condition on postoperative day 30. The timeline illustrating the diagnostic workup and therapeutic process is shown in Figure 4.

The patient recovered well at a follow-up visit 2 months after discharge. Echocardiography showed that the new aortic valve function remained excellent. No significant cardiovascular, pulmonary, or motor sequala were observed.

## Discussion

Severe AR is a medical emergency mainly caused by bicuspid aortic valve, endocarditis, rheumatic fever, and catheter-related injury. However, traumatic AVR after BCT is rarely encountered in the pediatric trauma population, and little attention has been paid to such damages (3, 6). In two retrospective studies of 237 pediatric patients with BCT over 5–9 years, no cardiac valve injury was reported (7, 8). The traumatic AR mechanism has been reported as a sudden increase of intra-thoracic pressure during the cardiac cycle's diastolic phase when the trans-aortic gradient is maximal, and this high pressure caused commissure dehiscence or aortic cusp rupture. NCC is most frequently involved due to no coronary artery buffers against hemodynamic stress (9), followed by RCC due to the closer position to the anterior chest wall may expose it to higher pressure. Intraoperative findings of complete disruption of the NCC and RCC but intact left coronary cusp in this patient agreed with the general injury patterns.

Traumatic AR should be diagnosed in time after BCT in the era of echocardiography, but early diagnosis (within 3 days) accounts for only 35%, while 43% were diagnosed after 1 month (5). Of the 5 pediatric cases reported, 2 had a significant heart murmur at the initial evaluation (10, 11), while 3 were found to have new heart murmurs or heart failure symptoms 7 days, 6 weeks, and 1 month after trauma, respectively (12–14). Valvular dysfunction can gradually worsen to symptomatic heart failure and results in significant morbidity and mortality if not diagnosed early.

Several factors may be responsible for the late detection of AVR in this patient (21 days after BCT). First, the poor acoustic windows due to the patient's body habitus and hemopneumothorax hindered an accurate evaluation of the cardiac valves by portable TTE (15, 16). The use of the S8-3 cardiac sector probe in this patient may also be an important reason for missed diagnosis because this type of cardiac probe may not obtain clear color Doppler blood flow signals in older children. Secondly, the presentation of acute AR after BCT may be insidious at the early stage but progress slowly, cardiac injury without immediate circulatory collapse may be masked by the consequences of multiple traumatic injuries. Finally, timely recognition of characteristic murmur of AR is challenging for non-cardiologists, especially in the setting of respiratory distress and tachycardia (17).

Patients with BCT should be repeatedly evaluated by physical examination and echocardiography to rule out cardiac injuries, especially valvular damage with an insidious onset and a slow progression. TEE should be performed to visualize intracardiac structures better if the images of TTE are suboptimal due to mechanical ventilation or other coexistent injuries (9). AVR should be highly suspected in patients presenting with widened pulse pressure, pulmonary edema, pulmonary hemorrhage, and diastolic heart murmur at the left lower sternal border. Elevated cardiac biomarkers including CKMB, CTnI, and NT-pro BNP also have a role in predicting cardiac injury. Early identification of AVR leads to an appropriate treatment strategy and improves prognosis. Aortic valve replacement, rather than repair, is the primary treatment modality because of avulsion injury or weakness of the remaining leaflet tissue, and the overall prognosis is good (5).

## Conclusion

This rare case highlights the importance of considering cardiac valve injury in patients with BCT, especially those presenting with signs and symptoms suggestive of heart failure. TEE should be performed if the findings of TTE are not clear.

## Data Availability Statement

The original contributions presented in the study are included in the article, further inquiries can be directed to the corresponding author.

## Ethics Statement

Ethical review and approval was not required for the study on human participants in accordance with the local legislation and institutional requirements. Written informed consent to participate in this study was provided by the participants' legal guardian.

## Author Contributions

FL was responsible for the diagnosis and treatment of the patient, Q-mZ and L-yL prepared the manuscript. LH collected clinic data. All authors read and approved the final manuscript and agree to be accountable for the content of the work. All authors contributed to the article and approved the submitted version.

## Conflict of Interest

The authors declare that the research was conducted in the absence of any commercial or financial relationships that could be construed as a potential conflict of interest.
